# Towards a standard benchmark for variant and gene prioritisation algorithms: PhEval - Phenotypic inference Evaluation framework

**DOI:** 10.1101/2024.06.13.598672

**Published:** 2024-06-16

**Authors:** Yasemin Bridges, Vinicius de Souza, Katherina G Cortes, Melissa Haendel, Nomi L Harris, Daniel R Korn, Nikolaos M Marinakis, Nicolas Matentzoglu, James A McLaughlin, Christopher J Mungall, David Osumi-Sutherland, Peter N Robinson, Damian Smedley, Julius OB Jacobsen

**Affiliations:** 1 William Harvey Research Institute, Barts and the London School of Medicine and Dentistry, Queen Mary University of London, London, EC1M 6BQ, UK; 2 European Bioinformatics Institute (EMBL-EBI), Hinxton, Cambridgeshire, CB10 1SD, UK; 3 School of Public Health, University of Colorado Anschutz Medical Campus, Aurora, CO, 80045, USA; 4 Department of Genetics, University of North Carolina, Chapel Hill, Chapel Hill, NC, 27599, USA; 5 Division of Environmental Genomics and Systems Biology, Lawrence Berkeley National Laboratory, Berkeley, CA, 94720, USA; 6 Laboratory of Medical Genetics, National and Kapodistrian University of Athens, Athens, 11527, Greece; 7 Semanticly, Athens, 10563, Greece; 8 Samples, Phenotypes, and Ontologies (SPOT), European Bioinformatics Institute (EMBL-EBI), Hinxton, Cambridgeshire, CB10 1SD, UK; 9 Wellcome Trust Sanger Institute, Hinxton, Cambridgeshire, CB10 1SA, UK; 10 Berlin Institute of Health, Charité – Universitätsmedizin Berlin, Berlin, 10117, Germany

## Abstract

**Background:**

Computational approaches to support rare disease diagnosis are challenging to build, requiring the integration of complex data types such as ontologies, gene-to-phenotype associations, and cross-species data into variant and gene prioritisation algorithms (VGPAs). However, the performance of VGPAs has been difficult to measure and is impacted by many factors, for example, ontology structure, annotation completeness or changes to the underlying algorithm. Assertions of the capabilities of VGPAs are often not reproducible, in part because there is no standardised, empirical framework and openly available patient data to assess the efficacy of VGPAs - ultimately hindering the development of effective prioritisation tools.

**Results:**

In this paper, we present our benchmarking tool, PhEval, which aims to provide a standardised and empirical framework to evaluate phenotype-driven VGPAs. The inclusion of standardised test corpora and test corpus generation tools in the PhEval suite of tools allows open benchmarking and comparison of methods on standardised data sets.

**Conclusions:**

PhEval and the standardised test corpora solve the issues of patient data availability and experimental tooling configuration when benchmarking and comparing rare disease VGPAs. By providing standardised data on patient cohorts from real-world case-reports and controlling the configuration of evaluated VGPAs, PhEval enables transparent, portable, comparable and reproducible benchmarking of VGPAs. As these tools are often a key component of many rare disease diagnostic pipelines, a thorough and standardised method of assessment is essential for improving patient diagnosis and care.

## Background

Rare diseases are defined as diseases affecting fewer than 200,000 individuals in the United States or fewer than 1 in 2,000 individuals in the EU [1]. It is estimated that over 400 million people worldwide are affected by some rare disease [2]. Oftentimes these diseases are so rare and complex that a patient may take years or even decades to receive an accurate diagnosis. Many rare diseases can be caused by extremely small errors in a patient’s genetic code. Accurately identifying genetic variants in patient data can greatly aid in the understanding of their conditions [3]. Since the average human has over 10 million variations in their genetic profile, most of which are not relevant to their disease(s), the process of utilising genomic information in diagnosis is twofold: identifying a specific patient’s variants by sequencing and then prioritising those variants to only those with a high likelihood of being relevant to the patient’s phenotypes of interest [4].

Next-generation sequencing (NGS) techniques, such as Whole Exome Sequencing (WES) and Whole Genome Sequencing (WGS), provide fast and cost-effective ways to quickly profile patient genetic information content. The extensive amount of genetic profiles generated by NGS techniques is then processed by variant and gene prioritisation algorithms (VGPAs). These algorithms enable clinicians and researchers to pinpoint potentially pathogenic variants responsible for rare diseases.

Phenotype data, which describes an individual’s observable traits, clinical features and medical history, plays a significant role in understanding the potential impact of genetic variants. The Human Phenotype Ontology (HPO) is a specially designed vocabulary and organisational structure which enumerates and categorises all human phenotypes [5]. HPO is widely used in diagnostic pipelines in many clinical and research settings.

In rare disease diagnosis, the integration of phenotype data into VGPAs has proven crucial for enhancing the accuracy and clinical relevance of genomic variant interpretation [6]. A key component of the integration of phenotype data and VGPAs is the utilisation of HPO terms, which serves as a crucial link between genomic information and clinical medicine, with the goal of covering phenotypic abnormalities in monogenic diseases [5]. More than 20 VGPA tools leverage HPO to incorporate phenotypic data in their algorithms [7], including well-known examples such as Exomiser[8], LIRICAL[9], and Phen2Gene[10]. Work by Robinson et al., Jacobsen et al., and Thompson et al. has provided insights into improving diagnostic yield achieved by integrating phenotype data within variant prioritisation algorithms [7, 11, 12].

Our group recently highlighted the importance of combining variant and phenotype scores into a unified score for the effective prioritisation of genetic variants [7] when utilising Exomiser[8], a VGPA developed by the Monarch Initiative[13]. The study demonstrated a significant advancement in the accuracy of Exomiser for predicting relevant disease variants when utilising a combination of genomic and phenotypic information. On a dataset of 4877 patients with a confirmed diagnosis and both genomic and phenotypic information, Exomiser correctly identified the diagnosis as the top-ranking candidate in 82% of cases, compared to only 33% and 55% when solely considering the variant or phenotype scores respectively. The incorporation of phenotype data from both mammalian and non-mammalian organisms into variant prioritisation algorithms has proven to increase accuracy even further. In work by Bone et al. the integration of diverse organism data, encompassing human, mouse, and zebrafish phenotypes, in conjunction with the phenotypes associated with mutations of interacting proteins, led to a substantial improvement of 30% in performance, with 97% of known disease-associated variants from the Human Gene Mutation Database (HGMD) detected as the top-ranked candidate in exomes from the 1000 Genomes project that were spiked with the causal variant [14].

There is a notable scarcity of benchmarks specifically designed for evaluating VGPAs. This hinders the objective assessment and comparison of different VGPAs. Without established benchmarks, researchers and clinicians encounter difficulties in gauging the accuracy, efficiency, and clinical relevance of different algorithmic approaches. The absence of these benchmarks also limits opportunities for identifying areas of improvement and innovation within the field of VGPA, potentially slowing down progress in rare disease diagnostics and genomic medicine as a whole.

Benchmarking VGPAs that rely on phenotype data presents a multifaceted challenge due to the extra algorithmic complexity required to monitor performance over time, as well as the additional requirements for test data, pre-processing and analysis. One key hurdle lies in the necessity to preprocess and transform the test data effectively. Patient phenotypic profiles are typically represented as a collection of HPO IDs (an alphanumeric code assigned to each specific phenotype term), and the descriptive label or name associated with each phenotype. However, the divergence in data formats expected by different VGPAs - from simple flat lists to highly structured - complicates the benchmarking process. GA4GH Phenopackets aim to provide a solution, serving as a standardised and extensible format for representing an individual’s disease and phenotype information, facilitating the consistent exchange of phenotypic data and playing a crucial role in genomics research by aiding in the understanding between genetic variations and observable traits [15].

The complexity of benchmarking these algorithms increases due to the variety of different interfaces that are needed to support these methods. Each algorithm must be individually invoked and executed, often demanding a significant amount of computational resources. Additionally, the logistical aspects of coordinating diverse software components with a variety of implementation details such as programming language and dependencies adds to the complexity. Even more complexity is created by the need to ensure the desired versions of each tool and input data version are used and to execute them in a systematic and reproducible manner.

Beyond the individual execution of algorithms, the benchmarking process also has to harmonise the diverse output formats generated by these tools. To enable meaningful comparisons and evaluations, the outputs must be transformed into a uniform format. This standardisation allows for consistent and structured analysis across different algorithms, ensuring that the results are interpretable and facilitating fair assessments of their performance.

To tackle the absence of standardised benchmarks and data standardisation for VGPAs, we developed PhEval, a novel framework that streamlines the evaluation of VGPAs that incorporate phenotypic data. PhEval offers the following value propositions:

- Automated processes: PhEval automates various evaluation tasks, enhancing efficiency and reducing manual effort.
- Standardisation: The framework ensures consistency and comparability in evaluation methodologies, promoting reliable assessments.
- Reproducibility: PhEval facilitates reproducibility in research by providing a standardised platform for evaluation, allowing for consistent validation of algorithms.
- Comprehensive benchmarking: PhEval enables thorough benchmarking of algorithms, allowing for well-founded comparisons and insights into performance.

### Implementation

PhEval is a framework designed to evaluate variant and gene prioritisation algorithms that incorporate phenotypic data to assist in the identification of possibly disease-causing variants. The framework includes (1) a modular library available on the Python Package Index (PyPI) repository of software that provides a command-line interface (CLI) to handle benchmarks efficiently, (2) an interface for implementing custom VGPA runners as plugins to PhEval, (3) a workflow system for orchestrating experiments and experimental analysis and (4) a set of test corpora.

### PhEval CLI and VGPA runners

The PhEval CLI comprises core and utility commands. The main command is “pheval run”, which executes the variant/gene prioritisation runners. Additionally, there are a collection of utility methods which facilitate some procedures such as generating “noisy” phenopackets to assess the robustness of VGPAs when less relevant or unreliable phenotype data is introduced (see Section on Test Corpora). The “benchmark” command allows users to evaluate and compare the performance of VGPAs algorithms by plotting a graph.

Another core component of PhEval is an extensible system to help enable the execution of a wide variety of VGPAs. To ensure that PhEval can execute and assess VGPA tools in a standardised manner, such tools must implement a set of abstract methods that ensure that (1) the data supplied by the PhEval framework is transformed into whatever input format the VGPA requires, (2) the VGPA tool can be executed using a standardised “run” method (described above, the main entry point for each benchmarking execution) and (3) the data produced by the VGPA tool is converted into the standardised representation required by PhEval to provide a comparison of performance of tools. All processes are described as part of the general PhEval documentation (https://monarch-initiative.github.io/pheval/developing_a_pheval_plugin/), and a concrete reference implementation is available at: (https://github.com/monarch-initiative/pheval.exomiser)

### PhEval experimental pipeline

The PhEval benchmarking process can be broadly divided into three distinct phases: the data preparation phase, the runner phase, and the analysis phase.

*The data preparation phase*, as well as automatically checking the completeness of the disease, gene and variant input data and optionally preparing simulated VCF files if required, gives the user the ability to randomise phenotypic profiles using the PhEval corpus scramble command utility, allowing for the assessment of how well VGPAs handle noise and less specific phenotypic profiles when making predictions. Additionally, PhEval offers some helper commands to produce and process semantic similarity profiles specifically for testing cross-species phenotype information in Exomiser. Phenotypic similarity is used in Exomiser to leverage information from both human and non-human species such as mice and zebrafish in the process of variant prioritisation in human cases [8]. PhEval seeks to make it easier to evaluate how different methods to compute phenotypic similarity, involving different algorithms and data sources, affect diagnostic yield. The development of the feature is in its early stages, and will be discussed in detail in a separate publication.

*The runner phase* is structured into three stages: *prepare*, *run*, and *post-process*. While most prioritisation tools are capable of handling some common inputs, such as phenopackets and VCF files, the *prepare* step plays a crucial role in adapting the input data to meet the specific requirements of the tool. For instance, one of the VGPAs we tested, Phen2Gene, which is a phenotype-driven gene prioritisation tool, lacks the ability to process phenopackets during its execution. To address this limitation, the *prepare* step serves as a bridge, facilitating necessary data preprocessing and formatting. In the case of Phen2Gene, potential solutions during the *prepare* step may involve parsing the phenopackets to extract the HPO terms associated with each sample, subsequently providing them to the Phen2Gene client in the run step. Alternatively, it may entail the creation of input text files containing HPO terms that can be processed by Phen2Gene.

In the *run* step, the VGPA is executed, applying the selected algorithm to the prepared data and generating the tool-specific outputs. Within the *run* stage, an essential task is the generation of input command files for the algorithm. These files serve as collections of individual commands, each tailored to run the targeted VGPA on specific samples. These commands are configured with the appropriate inputs, outputs and specific configuration settings, allowing for the automated and efficient processing of large corpora.

Finally, the *post-processing* step takes care of harmonising the tool-specific outputs into standardised PhEval TSV format, ensuring uniformity and ease of analysis of results from all VGPAs. In this context, the tool-specific output is condensed to provide only two essential elements, the entity of interest, which can either be a variant, gene, or disease, and its corresponding score. PhEval then assumes the responsibility of subsequent standardisation processes. This involves the reranking of the results in a uniform manner, ensuring that fair and comprehensive comparisons can be made between tools. PhEval offers an array of utility methods, readily available for integration into plugins by developers, facilitating the seamless generation of standardised PhEval outputs. These methods not only include the generation of the standardised output from extracted essential elements, but also methods for calculating the end position of a variant, converting gene names to specific gene identifiers and vice versa, contributing to an efficient post-processing workflow and ensuring the consistent and standardised representation of the results.

*In the analysis phase*, PhEval generates comprehensive statistical reports based on standardised outputs from the runner phase. This process enables rigorous assessment by comparing these results of VGPAs with the known causative variants (causal variants are provided in the set of phenopackets as an evaluation suite). These reports include both ranked metrics and binary classification. In our evaluation, true positives are defined as the known causative entity ranked at position 1 in the results, false positives are any other entity ranked at position 1, true negatives are any entity ranked at a position other than 1, and false negatives are the known causative entity ranked at a position other than 1. The ranked metrics we currently support calculating are: the count of known entities found in the top-n ranks, mean reciprocal rank, precision@k, mean average precision@k, f-beta score @k, and normalised discounted cumulative gain (NDCG) @k. The following binary classification metrics we support are: sensitivity, specificity, precision, negative predictive value, false positive rate, false discovery rate, false negative rate, accuracy, f1-score, and Matthews correlation coefficient. The framework currently offers robust support for analysing and benchmarking prioritisation outcomes related to variants, genes, and diseases, ensuring a thorough evaluation of its performance.

PhEval employs a Makefile (GNU-make) strategy to organise all necessary software execution. The GNU-make framework enables easy orchestration of the process of building data corpora, VGPA runs and benchmarking reports. In this framework, the researcher responsible for the benchmarking process can flexibly define recipes for every single data corpus, report, or run-configuration in a single file. During execution, results are cached in a way that specific recipes are only executed if any of its dependencies (like the corpus itself) has changed. PhEval provides a solution to create a Makefile dynamically. The file is generated using a Jinja template from configuration options listed in a YAML-based configuration file. This enables PhEval to systematically generate executable pipelines for all available combinations of runs as specified in the configuration file.

**Figure 1.**
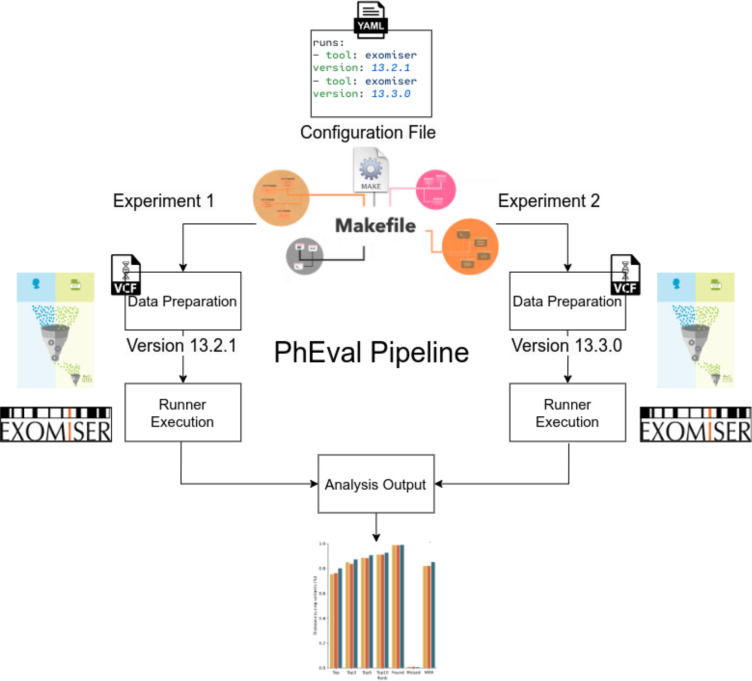
Pipeline workflow illustrative description from an experimental setting in an experimental setting where two distinct versions of Exomiser Runner (13.2.1 and 13.3.0) are compared. The workflow starts with the preparation of corpora for each experiment based on the YAML configuration file, which defines the parameters for the experiment. These corpora are then consumed by PhEval and Exomiser runners. Lastly, a consolidated report evaluating the results of the experiments is generated.

Figure 1 shows schematically how the framework ties the various phases of the benchmarking process together. First, the user provides a configuration file, which provides details on the exact algorithms used, comments on any modification that is being tested and version numbers. This configuration file is transformed into a Makefile which contains corresponding commands for experimental run configurations. Just before a run, the framework first ensures that the test data is appropriately prepared for a specific VGPA runner. Lastly, PhEval generates the analysis outputs for all configurations declared. An example run configuration can be accessed here: https://github.com/monarch-initiative/monarch_pheval.

### Test corpora in PhEval

#### Base corpus.

Our main test corpus is the “phenopacket-store” [16] by Danis et al. The corpus at the time of this writing comprised 4303 GA4GH phenopackets representing 277 diseases and 2872 unique pathogenic alleles, curated from 605 publications. Each phenopacket includes: a set of HPO terms describing the phenotypic profiles and a diagnosis, encompassing comprehensive information about the individuals previously documented in published case reports. This collection is the first large-scale, standardised set of case-level phenotypic information derived from detailed clinical data in literature case reports. Due to its size, we sometimes refer to the corpus as the 4K corpus in this paper.

#### LIRICAL corpus.

A small comparison corpus created for benchmarking the LIRICAL system [9] which contains 385 case reports. Variant information was generated by spiking causal genetic variants into a whole exome hg19 VCF file sourced from a healthy patient from the Genome in a Bottle dataset [17].

#### Synthetic corpus based on HPOA.

We also provide a corpus of 8,245 synthetically generated patients produced with *phenotype2phenopacket*. The synthetic corpus is created from the HPO annotations provided by the Monarch Initiative, specifically using the 2024–04-26 release. The primary objective of this corpus is to stimulate patient profiles specifically tailored to a specific disease. The corpus construction involves two steps, designed to represent the phenotypic characteristics associated with the disease while including noise.

HPO provides information on all possible phenotypes associated with that disease and the frequency at which they occur; we refer to this as the HPO term’s frequency value. In the first step of the corpus generation, a subset of HPO phenotypic terms is randomly selected for the disease, with the selection size varying between 20% and 75% of the total available terms. Each term undergoes scrutiny based on a randomly generated frequency value. If this value falls below the annotated frequency found in the HPO database, the term is deemed suitable for inclusion in the patient profile, to ensure diversity, terms lacking an annotated frequency are assigned a random frequency ranging from 0.25 to 0.75. This step also considers age onsets, where the patient’s age is generated within the range specified by the onset criteria from the HPO age of onset annotations.

Following the initial term selection, the profile is then further refined. In the next step, a subset of the selected terms is subjected to adjustments aimed at increasing or decreasing specificity. Each selected term undergoes a random number of adjustments within the ontology tree, involving movements up or down the tree by a specified number of nodes, ranging from 1–5. These adjustment steps are constrained to prevent terms from ascending beyond the top-level term “Abnormality of the X” in the HPO hierarchy. This adjustment process enriches the patient profiles with variability and specificity, aligning them with the complexity of real-world clinical scenarios.

#### Structural variantps corpus.

We provide GA4GH phenopackets which are used to represent 188 structural variants known to be associated with specific diseases, extracted from 182 case reports published in 146 scientific articles [18]. Structural variant information is generated by spiking causal genetic structural variants into a whole exome hg38 structural variant VCF from a healthy individual from the Genome in a Bottle dataset.

#### Phen2Gene corpus.

We provide 281 curated phenopackets using the primary data in the Phen2Gene benchmarking study [10]. This corpus represents individuals who were diagnosed with single-gene diseases and contain detailed phenotypic profiles as well as the known disease-causing gene.

#### PhEval Corpus Scramble Utility.

PhEval provides a “scramble” utility designed to randomise the individual phenotypic profile within an existing phenopacket. The term “scrambling” refers to the introduction of noise. This functionality is particularly valuable for assessing algorithm performance for prioritising variants and genes in the presence of noise. By applying different scramble factors on a corpus, the changes in performance can be observed. This approach stands in contrast to the synthetic corpus based on HPOA which generates a new phenopacket based on a disease and statistical information about phenotypic distribution of that disease. The scramble factor, specified in the range of 0–1, determines the extent of scrambling, with 0 indicating no scrambling and 1 indicating a complete randomisation of the phenotypic profile. The scrambling process uses a proportional approach for the generation of test data, leveraging existing patient data from a pre-existing corpus. It works as follows. Let **s** be a scrambling factor between 0 and 1 and let a given patient’s phenopacket have **n** phenotypic terms. The scrambling is performed by randomly selecting s * n of the phenotypic terms from the profile and modifying them, while leaving the unselected terms unmodified. The modification is as follows: s*n/2 or half of the selected terms are replaced by their parent term in the ontology, while the other half of the selected terms are replaced by another term in the ontology (with no concern for the original term).

During the process of scrambling, some terms are retained, others are converted to parent terms to decrease specificity, and additional random terms are introduced. Specifically, if a scramble factor of 0.5 was employed, half of the total number original phenotypic terms would be retained, regardless of the depth of the original term being replaced; a quarter of the total number would be converted to parent terms and the remaining quarter would be random terms added to the profile. This proportion based scrambling strategy accommodates the diverse lengths of phenotypic profiles, allowing researchers to systematically explore algorithm robustness under varied conditions.

## Results

In the following, we describe the outcomes of a benchmarking process that compares 2 versions of Exomiser, a modified version of Exomiser using updated semantic similarity mappings and scores for HPO to MP terms, a version of Phen2Gene and a version of GADO, see Table 1. We have selected this set of configurations to effectively illustrate the capabilities of PhEval, not to perform a comprehensive empirical study which would comprise dozens configurations. Therefore, for brevity, we omit details about the implemented tools and their configurations here but these are available from the GitHub repository as described below.

We tested all 5 configurations using the base corpus. The entire experimental setup, with the exception of the private corpora, is available at https://github.com/monarch-initiative/monarch_pheval.

**Figure 2A:**
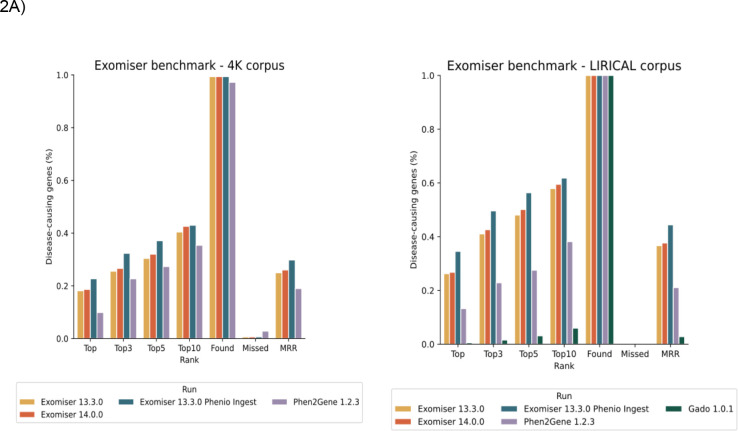

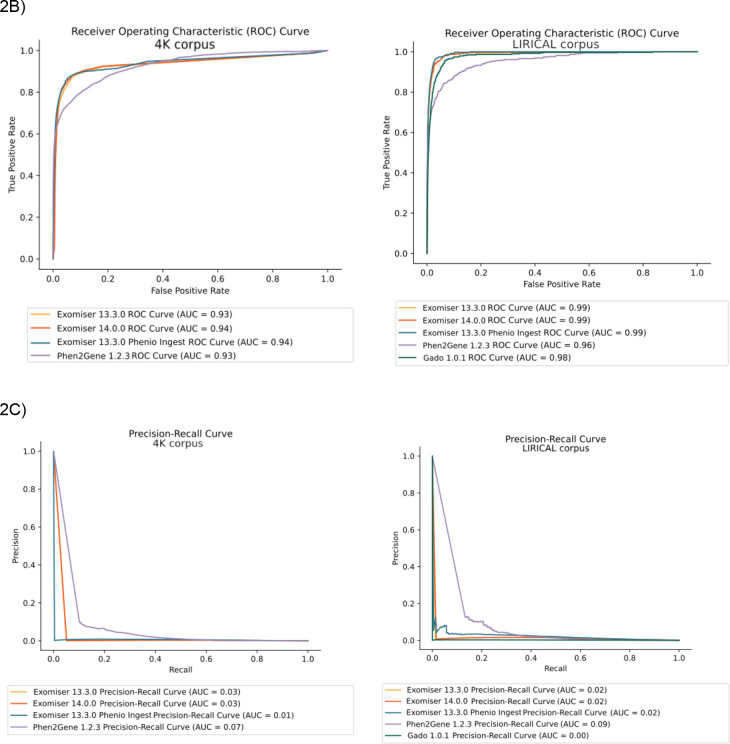
The bar plot shows the percentage of known disease-causing genes detected as the top ranked candidate, within the top 1, 3, 5, and 10 ranked candidates, as well as the percentage of found and missed genes. Lastly, MRR is a ranking metric whose value ranges from 0 to 1 where a larger value indicates that the top-ranked results are more accurate and relevant. Figures 2B (ROC curve) and 2C (Precision-Recall curve) depict statistical methods based on the confusion matrix of the causative genes. Note that GADO failed on the 4K corpus due to an internal error.

As can be seen by Figure 2A, the barplot demonstrates variations in performance across the evaluated VGPA configurations. First, we prepared the experiments for two corpora : the 4K corpus and the LIRICAL corpus. We carried out four experimental configurations using the 4K cohort and five experiments using the LIRICAL cohort. Among the evaluated configurations, Exomiser v13.3.0 with updated semantic similarity values notably outperforms the others, with a higher number of known causative genes captured in higher ranked outputs. An alternative approach to summarise the performance is to use ROC and Precision-recall curves, which depict the tradeoffs between true positive rate and false positive rate, as well as precision and recall, across different decision thresholds. In Figure 2B, all Exomiser versions demonstrate a steep ROC curve with a high area under the curve (AUC), indicating strong discriminative capacity and a high true positive rate at low false positive rates. Conversely, Phen2Gene 1.2.3 exhibits a less steep curve and lower AUC, suggesting a lower sensitivity and specificity. In the precision-recall analysis conducted against a 4K corpus, Exomiser experiments (14.0.0 and 13.3.0) started with high precision but dropped rapidly as recall increases (AUC = 0.03).

Similarly, Exomiser v13.3.0 with updated semantic similarity values achieves 0.01 as its AUC value. Finally, Phen2Gene experiment maintains the highest precision across all recall levels (AUC = 0.07). Despite the low AUC, Phen2Gene performed the best among the three models in precision-recall analysis, indicating its ability to identify relevant genes with more precision. This outcome was also observed when the experiments were conducted using the LIRICAL corpus, where Phen2Gene obtained the best result in precision-recall analysis again (AUC=0.09), while GADO had the poorest performance with an AUC of 0. Moreover, direct comparisons between different configurations were conducted to assess the impact of specific parameters or settings on ranking outcomes.. These comparisons illustrate how different configurations influence prioritisation outcomes, this analysis offers valuable insights into algorithm behaviour and performance under different conditions.

A complete breakdown of the generated metrics can be found in Supplementary Table 1 for LIRICAL corpus and Supplementary Table 2 for 4K (phenopacket-store) corpus.

## Discussion

Variant or gene prioritisation algorithms (VGPAs) are critical diagnostic tools, and as such should be benchmarked before they are utilised in healthcare. Numerous benchmarking studies have been conducted by researchers [14, 19, 20], primarily with the objectives of evaluating the performance of new prioritisation algorithms, performing comparative analyses with existing algorithms, and executing comprehensive reviews of algorithms already in use [9, 10, 21]. However, many of these benchmarks face challenges with reproducibility due to insufficient documentation of benchmarking methodologies. This makes it difficult to reproduce and validate results. Academic software providers and commercial vendors alike often make effectiveness claims, but these are infrequently validatable. A key step towards enhancing benchmark rigour involves providing transparent and detailed documentation, including data sources and versions, algorithm versions, data pre-processing steps, parameter settings, and provided uniform output from each tool. An anecdote that illustrates the importance of clearly documenting benchmarking methodologies and providing the experimental pipeline in a reproducible manner is demonstrated by a comparative analysis of ten gene-prioritisation algorithms performed by Yuan et al. The study revealed a significant variance in Exomiser’s performance compared to previous benchmarking outcomes [22]. In response, Jacobsen et al. clarified this discrepancy by identifying crucial differences in the parameter settings employed by Yuan et al., which were the likely explanation for the observed differences in the performance of Exomiser [23]. Fortunately Yuan et al. included the data necessary for reproducing their study, which played a pivotal role in facilitating the correction of discrepancies.

Beyond concerns related to documentation and reproducibility, another significant issue in present benchmarks is the lack of standardisation, particularly concerning the types of test data and performance evaluation metrics used. Consequently, some test datasets may exhibit better performance than others, and the choice of which metrics to use may favour specific algorithms, introducing an element of subjectivity. Such variability raises questions about the consistency and comparability of benchmark results across studies. Additionally, while synthetic datasets are valuable tools for controlled evaluations, they come with inherent limitations in their ability to represent real-world scenarios. Simulated datasets are typically designed based on simplified models, and as such, may not encompass the full spectrum of genetic variants and phenotypic complexities encountered in clinical settings. The use of simulated data may inadvertently favour algorithms that perform well under the specific conditions and assumptions embedded in these datasets, potentially leading to results that diverge significantly from those observed in real-world applications. These challenges collectively create conditions that can make achieving rigour in benchmarking studies more difficult.

Existing benchmarks predominantly report recall-based metrics, often measuring the algorithm’s capability to capture all relevant candidates in a reasonable set of ranked candidates. For example, the Phen2Gene benchmark assessed the performance of Phen2Gene against three other gene prioritisation tools. In this benchmark, researchers compared the number of prioritised genes that were found in the top 10, 50, 100 and 250 ranks for solved cases [10]. The concentration on recall metrics only provides a partial view of the algorithm’s performance and may inadvertently encourage algorithms to generate longer lists of equally ranked candidates, increasing the chances of identifying crucial genes or variants in the top X hits. However, a recall-centric approach can also lead to a higher rate of false positives (low precision), which may not always align with the demands of diverse research contexts, e.g., under-resourced diagnostic laboratories that can only properly interpret a handful of variants per case.

This perspective highlights the need for a balanced evaluation that takes both precision and recall into account when evaluating VGPAs for diagnostic use; as well as other metrics which may aid in the fine-tuning of algorithm processes. As reported in our results, we have provided a comprehensive evaluation using both ranking and binary statistics to demonstrate the performance of different configurations.There are existing benchmarks that go beyond the confines of recall metrics and provide a better picture into algorithm performance. For example, other efforts have explored insights into the sensitivity and specificity of algorithms by using area under the curve (AuC) from Receiver Operating Characteristic (ROC) in addition to an algorithm’s precision[11, 24, 25]. However, it can be difficult to draw direct comparisons between benchmarks that report different metrics due to the variations in the level of detail provided by these insights highlighting the need for the standardisation of an evaluation process and furnishing a consistent set of metrics and evaluation protocols.

PhEval was designed to provide a transparent, easy to use experimental framework that addresses issues such as the one illustrated by the anecdote above. A representation of the experimental design in a single configuration file, a fully transparent executable pipeline in a known data orchestration format (GNU-make) and standardised runner configurations and versioned and standardised test data are key features that increase the transparency and reproducibility of VGPA benchmarks.

Standardising the benchmarking process of VGPAs is complicated further by the increasing need of leveraging phenotype data alongside more classic gene-focused methods[26]. Modern VGPAs increasingly rely on phenotype data to improve diagnostic yield. An example of a VGPA tool that leverages cross-species phenotype data is Exomiser. Exomiser has also played a pivotal role in numerous projects and pipelines dedicated to novel gene discovery. Leveraging organism phenotype data proves to be an important step in the context of functional validation, as demonstrated by Pippucci et al., who used Exomiser to enhance the prioritisation of candidate genes in a case of epileptic encephalopathy, ultimately identifying a previously undiscovered mutation in CACNA2D2 to be causative of the disease [27]. PhenomeNET Variant Predictor (PVP), an alternative variant prioritisation algorithm, has also shown that the incorporation of mouse and fish phenotype data is especially useful in instances where human phenotypic information for a specific gene is lacking. Notably, PVP found substantial improvements in variant ranking when incorporating organism phenotype data in comparison to human alone. In a specific example genomic information relating to Hypotrichosis 8 was screened by PVP, initially variant rs766783183 present in the gene KRT25 was ranked at 172 (without the inclusion of model organism data); when this data was included this variant’s prioritisation ranking significantly improved to rank 8, this variant was ultimately the confirmed molecular diagnosis for Hypotrichosis 8[28]. Utilising model organism phenotype data thus emerges as a pivotal strategy, not only in enhancing variant prioritisation algorithms but also in advancing novel gene discovery methods. This evidenced in our results, where Exomiser, the only VGPA incorporating model organism phenotype data in our experiments, outperformed both Phen2Gene and GADO in capturing the number of genes within the Top N hits.

Systematic benchmarking of VGPAs is important to monitor diagnostic yield in an environment that involves complex interactions between phenotype data and algorithms, but no standardised frameworks exist that support the entire benchmarking lifecycle. The closest one is VPMBench[29] which automates the benchmarking of variant prioritisation algorithms, but not the benchmarking of VGPAs that specifically leverage phenotype data. Leveraging phenotype data not only increases the complexity of the algorithms and therefore the importance of systematic benchmarking to monitor performance over time. It also increases the complexity of the evaluation itself, because of additional requirements on test data, test data pre-processing and analysis. PhEval has been designed to standardise the evaluation process for VGPAs with a specific focus on algorithms that leverage phenotype data. As we can see in Figure 2 the standardisation of analytical results supported by rigorous statistical methods, enables straightforward comparisons among various VGPAs. PhEval is built on phenopackets, a GA4GH and ISO standard for sharing detailed phenotype descriptions with disease, patient, and genetic information, enabling clinicians, biologists, and disease and drug researchers to build more complete models of disease. Individual phenopackets correspond to case descriptions that contain critical information that can inform the VGP process. Every test in PhEval corresponds to a phenopacket, which not only ensures that every test case is appropriately standardised, but also that future test data that is already standardised as phenopackets can be seamlessly integrated into PhEval without complex transformation pipelines.

The trend towards ever more complex data-algorithms interactions is likely to accelerate with the dawn of Large Language Models (LLMs). We have already started experimenting with PhEval runners that leverage LLMs (https://github.com/monarch-initiative/pheval.ontogpt).

Such VGPA approaches are not only likely to be sensitive to the exact data presented for the contextualisation of a case description - their current propensity to hallucination requires carefully designed mitigation strategies and careful monitoring in the form of repeated benchmarking to ensure that they perform appropriately in a diagnostic scenario[30].

To facilitate wide uptake of the framework for experimental studies, an easy way to integrate existing VGPAs with often idiosyncratic distributions, configuration requirements and technology dependencies is needed. PhEval provides an easy-to-implement system to integrate any runner, which requires the implementation of a handful of methods; see Section on the Implementation of the PhEval CLI and VGPA runners. The instructions we provide for implementing runners such as these(https://monarch-initiative.github.io/pheval/developing_a_pheval_plugin/) also include a simple way to enable their publication on PyPi. This way, other experimenters can simply install runners that have already been implemented, like Exomiser, Phen2Gene and X, e.g. “pip install pheval.exomiser”.

## Limitations

### Corpus bias.

The most significant limitation of any specific framework for assessing the performance of diagnostic tools is the lack of publicly available real clinical data. This is no different in the case of PhEval. In practice, we execute PhEval on a number of private corpora such as the rare disease component of the 100,000 Genomes Project in the Genomics England (GEL) research environment, diagnosed cases from the Deciphering Developmental Disorders (DDD) project [31], and a retinal cohort [32].

The lack of a gold standard complicates comparative analyses among different algorithms. Variant prioritisation algorithms typically depend on curated databases of known disease-associated variants including specific subsets categorised by their established clinical significance, such as pathogenic or benign variants. These databases of curated disease-gene or disease-variant relationships, for example HPO and ClinVar, are often used for benchmarking [5, 33]. While these datasets serve as valuable resources for benchmarking, they are limited in scope and may not fully represent the genetic variability and diseases encountered in real-world scenarios. Specifically, the phenotype data typically presents as a merge of all possible phenotypes for a disease (HPOA) or a disease label (ClinVar) resulting in a loss of specific individual phenotype information that could otherwise be linked to genetic variants. In light of these challenges, it becomes imperative for the research community to work collectively toward establishing standardised benchmarking methodologies and datasets to advance the ability to openly and rigorously evaluate and compare VGPAs.

While the development of a proper representative gold standard corpus of real clinical samples is still largely out of sight, we are constantly working on increasing our public test corpora. We are currently building a phenopackets store (https://github.com/monarch-initiative/phenopacket-store), which contains an extensive collection of Phenopackets that represent real individuals with Mendelian disease reported in case reports in the literature.

## Conclusions

Variant prioritisation is critical for diagnosis of rare and genetic conditions at scale. To effectively support diagnostics, VGPA methods increasingly need to leverage all the available data, in particular phenotype. As our ability to leverage phenotype data in more sophisticated ways increases, for example by including gene-to-phenotype associations from across different species, the complexity of the system increases as well. To ensure accurate evaluations of VGPA tools and monitoring their performance across versions, robust methods must be developed to evaluate the complex interplay between algorithms and data. PhEval is the first framework that takes phenotype data directly into account during the VGPA benchmarking process by standardising input data as GA4GH phenopackets.

## Supplementary Material

Supplement 1

Supplement 2

## Figures and Tables

**Table 1:** VGPA tools evaluated as part of the experiment.

VGPA Tool	Version	Features
Exomiser	13.3.0	Release version
Exomiser	14.0.0	Release version
Exomiser	13.3.0.b	Experimental version with updated semantic similarity values
Phen2Gene	1.2.3	Release version
GADO	1.0.1	Release version

## Data Availability

The package source code repository can be accessed via GitHub at https://github.com/monarch-initiative/pheval. The documentation can be found at https://monarch-initiative.github.io/pheval/. The data used to produce the results in the study can be found at https://zenodo.org/records/11458312 and the code used for reproducing the benchmarking from this paper is available at https://github.com/monarch-initiative/monarch_pheval. The PhEval corpora are under constant development and can be found at https://github.com/monarch-initiative/pheval/tree/main/corpora.
